# Prophylaxis of thromboembolic disease: patient safety protocoL

**DOI:** 10.1186/2197-425X-3-S1-A736

**Published:** 2015-10-01

**Authors:** R Viejo Moreno, M Talayero Giménez de Azcárate, J Barea Mendoza, H Domínguez Aguado, N Regueiro Díaz, L Díaz Castellano, M Castillo Jaramillo, LJ Terceros Almanza, S Temprano Vázquez, J Á Sánchez-Izquierdo Riera, JC Montejo González

**Affiliations:** 12 de Octubre University Hospital, Intensive Care Medicine Department. Medical Intensive Care Unit, Madrid, Spain

## Introduction

Critically ill patients have a high risk of deep venous thrombosis and pulmonary embolism, which comprise venous thromboembolic disease (VTED). Prevention during critical illness is a widely used quality metric and safety initiative for these patients.

## Objective

To evaluate the effectiveness of a bundle of VTED prophylactic measures upon completion of a project aim to improve patient safety in this entity.

## Methods

We designed a three phases prospective study of patients admitted to a general ICU of a tertiary university hospital. During first 4 months, weekly collection days are established, gathering demographic variables, reason for admission, severity scores, risk factors for hemorrhage and thrombosis as well as VTED prophylactic measures prescribed. Data were analyzed using a Failure Mode and Effects Analysis (FMEA) from which a set of measures were developed and the implementation of a VTED prophylaxis protocol. After that, we collected data during 5 months to compare the effectiveness of the protocol. Finally, a checklist was introduced to facilitated the adherence to these measures. The impact of this checklist was evaluated for 2 more months. All results were analyzed using the SPSS v22.0.0 statistical analysis software.

## Results

In the first period we enrolled 59 patients, 42 of them (71.2%) received prophylaxis for VTE (63.4% had pharmacological prophylaxis and 34.1% mechanical) and 2.4% received dual prophylaxis. Seventeen patients (28.8%) received no prophylaxis, 2 of them had contraindication to any type of VTED thromboprophylaxis.

Post-FMEA, we enrolled 97 patients, 89 of them (91.7%) received prophylaxis for VTED: 55% received pharmacological prophylaxis and 45% mechanical devices. Dual prophylaxis was received by 6.25% of patients. From those who received mechanical devices, 67.9% received compression stockings. Seven patients (8.2%) presented high risk of bleeding and did not receive prophylaxis. One patient had contraindication for both types of measures.

Post checklist we included 40 patients, 39 of them (97.5%) of them received prophylactic measures: 73.5% received pharmacological prophylaxis and 32.5% mechanical devices. Dual prophylaxis were applied in 25% of patients. Only 1 patient (2.5%) had double contraindication for VTED measures.

## Conclusions

The number of patients with high risk of thrombosis as well as those who receive dual prophylaxis has increased after the implemetation of a VTED prophylaxis protocol. The development of a daily checklist could be a useful tool to monitor adherence to this protocol. All of these measures are expected to improve patient safety.

